# DNA‐based identification of anadromous fishes (*Alosa* spp., Family Clupeidae) in stomach contents of marine groundfish

**DOI:** 10.1111/jfb.70415

**Published:** 2026-03-31

**Authors:** Landon P. Falke, Stacy Rowe, Yuan Liu, Timothy F. Sheehan

**Affiliations:** ^1^ Azura Consulting LLC Contractor for NOAA's Northeast Fisheries Science Center Woods Hole Massachusetts USA; ^2^ NOAA, National Marine Fisheries Service Northeast Fisheries Science Center Woods Hole Massachusetts USA; ^3^ Ocean Associates Inc Contractor for NOAA's Northeast Fisheries Science Center Milford Connecticut USA; ^4^ Gloucester Marine Genomics Institute Gloucester Massachusetts USA

**Keywords:** anadromous prey, DNA barcoding, prey identification, stomach content analysis

## Abstract

Using DNA techniques for prey identification is an emerging approach for enhancing the precision and accuracy of trophic information. We evaluated the effectiveness of DNA‐based prey identification in conjunction with visual stomach content analysis of commercially important groundfish in the nearshore Gulf of Maine, with a focus on distinguishing consumed anadromous (*Alosa* spp.) and closely related marine prey (Atlantic herring *Clupea harengus*, Atlantic menhaden *Brevoortia tyrannus*). DNA barcoding of 179 consumed prey specimens provided species‐ or genus‐level identification in 122 cases (68.2%), including 104 improvements to taxonomic resolution and three conflicting results compared to visual identifications. Combining DNA‐based identifications of partially or well‐digested prey with the broader visual analysis substantially improved quantification of a diet metric, frequency occurrence, as a proxy for the relative diet contributions of anadromous and marine clupeids. Body size measurements of consumed *Alosa* spp. identified using DNA were consistent with year‐0 or year‐1 juvenile life stages. Possible limitations to success rates of DNA assignment included the quality of sample preservation (ethanol), reference database quality (accuracy of voucher specimen identification) and sequencing errors (miscalling of nucleotide bases). Despite this, the integration of molecular methods substantially improved the interpretability of trophic interactions involving morphologically similar prey with ecologically important differences in life history. These results highlight how the targeted application of DNA‐based prey identification can complement conventional diet analyses by improving the quantification of trophic interactions that ultimately govern ecosystem processes.

## 1 INTRODUCTION

Stomach content analysis is the most commonly used approach for fish diet assessments despite limitations in identifying partially digested prey (Amundsen & Sánchez‐Hernández, 2019; Hynes, 1950; Hyslop, 1980). The traditional approach to stomach content analysis involves visual identification of prey using diagnostic morphological characters. A major advantage of this approach over indirect diet assessments (e.g. fatty acid analysis, isotope analysis) is that the direct examination of stomach contents can provide precise information on the amount, sizes and life stages of consumed prey (da Silveira et al., 2020; Nielsen et al., 2018). However, the degradation of diagnostic characters during mastication and digestion often limits the taxonomic resolution of prey identification, especially when stomach contents consist of morphologically similar species and/or early life stages of prey (Buckland et al., 2017; da Silveira et al. 2020; Hyslop, 1980; Legler et al., 2010; Schooley et al., 2008). As a result, diet data derived from traditional stomach content analyses often involve coarse taxonomic resolution, incomplete prey size and life‐stage information, and digestion‐related biases that can undermine its utility.

The application of DNA‐based prey identification is a promising approach for improving the specificity and accuracy of information gained from stomach contents. The major advantage of using DNA is that it can provide verifiable, often species‐level identification, even if prey are well‐digested and only small amounts of tissue remain (e.g. Dunn et al., 2010; Jarman et al., 2002; Thalinger et al., 2016). DNA‐based identification of species follows a standardized protocol in which a segment of a diagnostic gene is amplified, sequenced and compared against a reference database of sequences derived from known species, a process commonly referred to as DNA barcoding (Hebert et al., 2003). Mitochondrial DNA cytochrome c oxidase 1 (COI) is often used as the diagnostic molecular marker because its high interspecific and low intraspecific variability allow for efficient species‐level identification among closely‐related animals (Hajibabaei et al., 2006; Hebert et al., 2003; Waugh, 2007; Weigt et al., 2012). The development of universal COI fish primers and the compilation of COI sequences from thousands of fish species in public databases (e.g. GenBank [Benson et al., 2004], Barcode of Life Database [Ward et al., 2009]) have provided a standardized foundation for using DNA methods to improve diet assessments involving fishes as predators and prey (reviewed by Traugott et al., 2021). Despite these advantages, DNA‐based identification of partially digested prey involves its own challenges and limitations. Confident species identification using a DNA sequence relies on the completeness and accuracy of reference databases and on successful DNA extraction and amplification from degraded material (Berry et al., 2015; Deagle et al., 2007; Leray et al., 2013; Pentinsaari et al., 2020). The analytical demands of DNA barcoding can also limit its scalability compared to simpler morphological approaches. Rather than applying either approach in isolation, comparative studies suggest that integrating morphological and molecular approaches might be the best solution for maximizing prey identification rates and ecological insights in diet assessments (Aguilar et al., 2017; Berry et al., 2015; Casper et al., 2007; Oehm et al., 2017; Tollit et al., 2009).

The present study applies DNA barcoding to facilitate the identification of anadromous fishes (*Alosa* spp., Family Clupeidae) in the stomach contents of marine groundfish. Fishes in the genus *Alosa* include alewife *Alosa pseudoharengus* (Wilson 1811), blueback herring *Alosa aestivalis* (Mitchill 1814) and American shad *Alosa sapidissima* (Wilson 1811). The anadromous life history of *Alosa* spp. involves seasonal migrations to and from the mouths of freshwater drainages (Acolas & Lambert, 2016). Predator–prey interactions involving anadromous fishes in marine food webs are generally not well understood, but the small body sizes of *Alosa* spp. make them viable prey for a wide variety of marine predators (Jones et al., 2010; Juanes et al., 1993; Leach et al., 2024; McDermott et al., 2015; Smith & Link, 2010; Toth et al., 2018). However, abundances of *Alosa* spp. have declined throughout their range, primarily due to reduced access to freshwater spawning and rearing habitats (Hall et al., 2011; Limburg & Waldman, 2009). Large‐scale restoration efforts, such as dam removals or fish passage engineering, aim to re‐establish migratory pathways to support recovery of anadromous species (Hall et al., 2011, 2012). This has increased interest in quantifying diet contributions of anadromous prey in nearshore food webs (Falke et al., 2024; McDermott et al., 2015; Willis et al., 2017). However, quantifying the diet contributions of *Alosa* spp. prey is challenging due to the limited taxonomic resolution in conventional visual analyses. *Alosa* spp. can co‐occur in diets with morphologically similar, purely marine clupeids (Falke et al., 2024), including Atlantic menhaden *Brevoortia tyrannus* (Latrobe1802) – which share the subfamily Alosinae with *Alosa* spp. – and Atlantic herring *Clupea harengus* L. 1758. Diet metrics calculated at sub‐family, family or lower‐resolution levels can therefore overlook important ecological differences among these prey species and their life stages. Higher‐resolution identification enabled by DNA techniques is therefore crucial for advancing trophic ecology and ecosystem management related to anadromous fishes.

Our primary aims were to assess the effectiveness of DNA‐based prey identification of potential *Alosa* spp. prey in stomach contents, examine factors influencing barcoding success and evaluate how integrating visual‐ and DNA‐based identifications improve calculations of a quantitative diet metric. To address these objectives, we applied DNA barcoding to visually ambiguous clupeid prey recovered from marine groundfish stomach contents and integrated molecular results with conventional visual identifications. We hypothesized that digestion state and preservation (sample age) would significantly influence barcoding success rates, but expected DNA barcoding to improve the taxonomic resolution of prey identifications overall compared to visual‐only methods. Thus, we expected that combining visual identifications and targeted DNA analyses would shift diet metrics toward higher‐resolution prey categories, thereby enhancing ecological insights into the role of anadromous fishes in marine food webs.

## 2 MATERIALS AND METHODS

### 2.1 Ethics statement

The care and use of experimental animals complied with US federal animal welfare laws, guidelines and policies. All sampling procedures were conducted in accordance with protocols approved by NOAA's Northeast Fisheries Science Center. Stomach sampling was performed as part of the Maine–New Hampshire Inshore Trawl Survey (MEDMR, 2023), and was supported by NOAA Grant/Award Number: NA22NMF4540361.

### 2.2 Sample area and field collections

**FIGURE 1:**
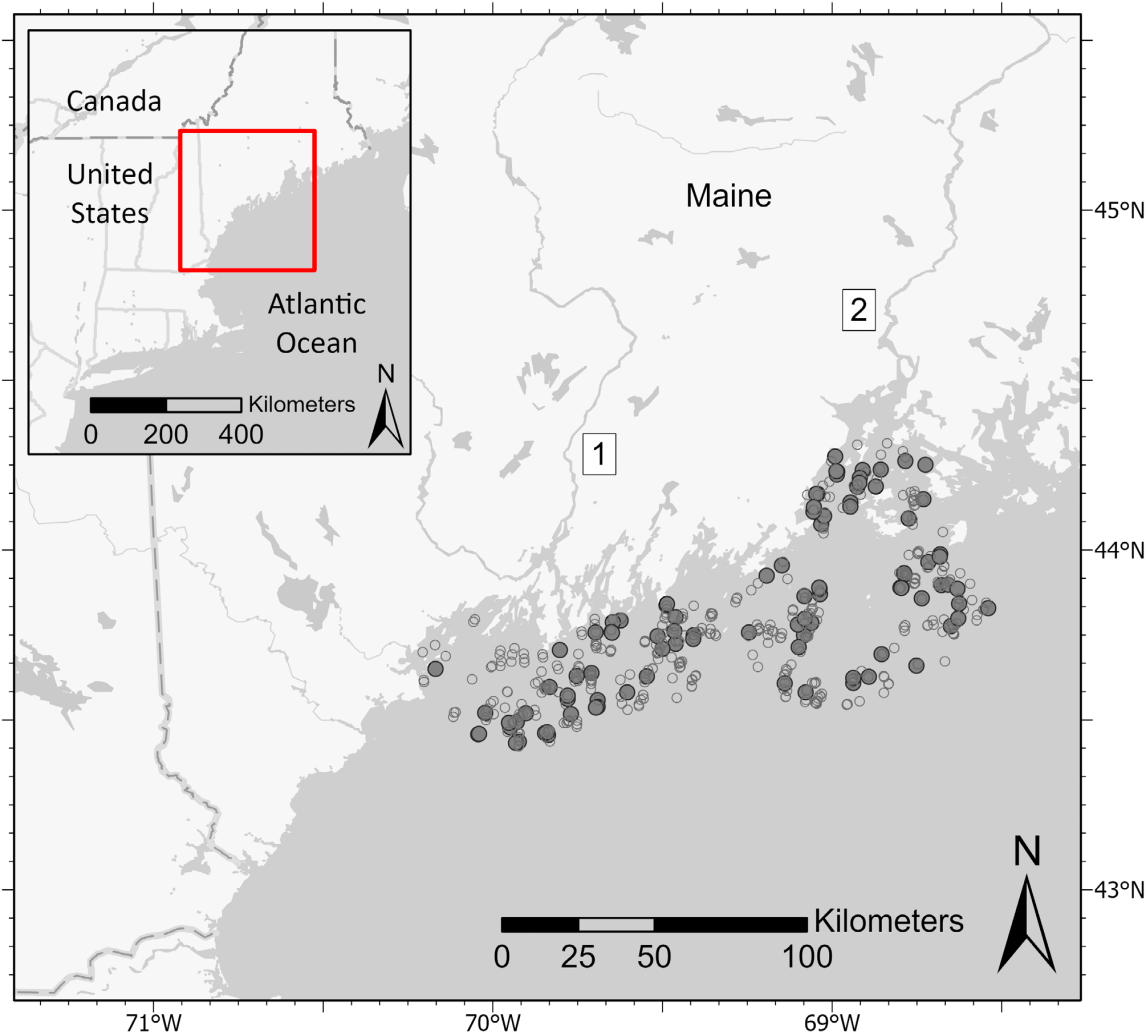
Map of field collection locations of prey specimens subjected to DNA analysis (filled circles) found in groundfish stomachs in the nearshore Gulf of Maine. Smaller, unfilled circles show all other locations where groundfish stomachs were sampled during the 2012–2022 diet study. The sample area was located in proximity to two major freshwater drainages of Maine: (1) Kennebec River and (2) Penobscot River. Inset map shows the location of the sample area (outlined in red) in the northeastern United States.

Bottom trawls were used to collect silver hake *Merluccius bilinearis* (Mitchill 1814), Atlantic spiny dogfish *Squalus acanthias* L. 1758, monkfish *Lophius americanus* Valenciennes 1837, white hake *Urophycis tenuis* (Mitchill 1814) and red hake *Urophycis chuss* (Walbaum 1792) from nearshore marine waters in the Gulf of Maine during bi‐annual survey periods (spring and fall) from 2012 to 2022 (Figure 1). Areas near the mouths of the Kennebec and Penobscot Rivers were chosen for sampling by McDermott et al. (2015) because of the contemporary anadromous fish populations and ongoing restoration history in these rivers. Stomach sampling focused on potential predators of piscine prey with a minimum centreline length of 15 cm (*L. americanus*) or 20 cm (*M. bilinearis*, *U. chuss*, *U. tenuis* and *S. acanthias*). Whole stomachs were removed and preserved with 95% ethanol on board the fishing vessel. Falke et al. (2024) provide additional details on sampling design for the 2012–2022 diet study.

### 2.3 Laboratory processing of stomach contents

All contents were removed from each stomach and prey were assigned a best‐possible taxonomic identification based on visual assessment of morphological features using microscopy (6× to 50× magnification). Morphological features used for visual identification of clupeids included otolith shape, presence/absence of ventral scutes, pigmentation of the peritoneum, rib cage structure and body shape (e.g. dorsal‐ventral width, eye size). Actual or estimated total length was recorded for intact piscine prey. All piscine prey were rated as fresh (few signs of digestion, external characteristics typically intact), partially digested (prey softened and exterior eroded, skin mostly gone but majority of tissue still present) or well‐digested (external characteristics of prey missing, identification relying on internal characteristics and hard parts; Figure S1). Stomach contents were returned to 95% ethanol prior to DNA processing.

### 2.4 Sample selection and DNA extraction

Based on their identification as a possible *Alosa* spp. (e.g. sub‐family Alosinae, unclassified Clupeidae, unidentified bony fish or a species‐level assignment needing confirmation), 179 piscine prey specimens were targeted for DNA analyses conducted in 2020 (*n* = 93) and 2023 (*n* = 86). The unidentified bony fishes selected for tissue extraction were those that showed potential for being Alosinae; bony fishes that did not show potential for being Alosinae were not selected due to cost and time restraints. Sample selection prioritized samples collected closest to the date of DNA analysis and proceeded in reverse chronological order until processing capacity was reached. Extremely degraded samples (e.g. chyme, particulates, bones) were not selected. Samples with DNA analysis in 2020 were collected from surveys in 2019 (*n* = 39), 2018 (*n* = 37) and 2017 (*n* = 17). Samples with DNA analysis in 2023 were collected from surveys in 2022 (*n* = 23), 2021 (*n* = 51) and 2019 (*n* = 12). In 2023, muscle tissue samples from positively identified, trawl‐caught *A. pseudoharengus* (*n* = 2), *A. aestivalis* (*n* = 2) and *A. sapidissima* (*n* = 1) from the same survey area were analysed using the same DNA protocol to provide a control for DNA‐based identification of anadromous taxa. All positive control samples were collected 2 years before the DNA analysis.

Prior to DNA extraction, 200–400 mg of muscle tissue was excised from each specimen. To avoid cross‐contamination between samples, disposable plastic trays and/or non‐absorbent cellulose weighing papers were used as work surfaces, and dissection tools (scissors, knives, scalpels, forceps) were cleaned and sterilized with ethanol. Severely degraded and surface tissues were avoided when possible. Samples were placed in 2 mL Eppendorf tubes and a wash was performed to remove ethanol by aliquoting 1 mL of 1XPBS buffer to each tube and centrifuging at 8500*g* for 4 min followed by the removal of supernatant. The wash was repeated once, after which each clean and dry tissue sample was placed in a new 2 mL Eppendorf tube and frozen until extraction. Extraction was performed following Quick‐Start Protocol for QIAamp® Fast DNA Stool Mini Kit (Qiagen). After extraction, quantification using Qubit dsDNA Quantification, High Sensitivity Assay kit was performed. Fifty microlitres of sample aliquots were placed into 0.1 mL sterile PCR tube strips and frozen in preparation for PCR and Sanger sequencing, which was conducted at the Northeastern University Ocean Genome Legacy Center.

### 2.5 DNA amplification and sequencing

Approximately 700 bp of the mitochondrial cytochrome oxidase I (COI) region was amplified using tailed M13 COI primers FISHCOILBC_ts and FISHCOIHBC_ts (Handy et al., 2011). For each PCR reaction, 2 μL of DNA template (2 μL of H_2_O for negative controls), 17.5 μL of OneTaq 2X Master Mix (New England Biolabs) and 10 μM of both forward and reverse primers were mixed together and brought up to 35 μL total volume with MilliQ water. The thermal cycle included a hot start at 94°C for 30 s, 30 cycles of 94°C for 30 s, 52°C for 40 s and 68°C for 60 s, and a final extension at 68°C for 5 min using a PCT‐200 thermocycler (MF Research, Inc.). PCR success was visualized by 0.8% agarose gel electrophoresis. Negative controls did not produce visible bands on agarose gels and were therefore not sequenced.

From each amplicon, 15 μL was bi‐directionally sequenced on an Applied Biosystems 3730xl DNA Analyser at Psomagen (Rockville, MD). Resulting sequences were edited and analysed using Geneious v.8, automatically trimming ends to remove sequences with greater than a 1% chance of error per base and setting 500 bases as a minimum threshold for a successful read.

Species identifications were determined using BLAST against the FDA References Standard Sequence Library for seafood identification, as well as the Barcode of Life Data Systems (BOLD) and National Center for Biotechnology Information (GenBank) databases. Species was only assigned when there was a >99% match between query and reference sequences. When query sequences matched with multiple reference species, the lowest common taxon‐level was assigned.

### 2.6 Statistical analysis

Logistic regression analysis was performed to verify the effects of prey size and digestion state on the resolution of piscine prey identification in the visual stomach content analysis (i.e. not including DNA‐based prey identifications). All observations of intact piscine prey in groundfish stomach contents over the 2012–2022 survey period (*n* = 1202) were included in this analysis. A generalized linear model and logit link function were used to model a binary response (1 = a species or genus‐level identification was made, 0 = a lower‐resolution identification was made). Piscine prey size (total length in mm) and digestion state were included as additive predictor variables. Digestion state was considered a two‐level categorical variable (fresh/partial and well‐digested) in this model because all prey classified as ‘fresh’ were given a species‐level identification using visual methods.

Logistic regression was also used to examine the effects of sample age and digestion state on the success of DNA‐based prey identification of the 179 selected specimens. A generalized linear model and logit link function were used to model success/failure of DNA‐based identification (i.e. yielding a species‐ or genus‐level match to reference database sequences). Sample age (continuous; time in years between initial sample preservation and DNA extraction) and digestion state (three‐level categorical variable; fresh, partial digestion or well‐digested) were included as predictors in the model. The significance of a two‐way interaction between sample age and digestion state was first determined using a likelihood ratio test (LRT), in which the full model with the two‐way interaction was compared to a reduced additive model with main effects only. After determining the two‐way interaction was not significant, an additive model was used to determine the main‐effect significance of sample age and digestion state. Because predator species were not expected to explain the probability of successfully identifying prey, data were pooled across the five predator species, although the majority of stomach content samples were from *M. bilinearis*. Maximum likelihood estimation was used to calculate coefficients and standard error, and Wald tests were used to test the significance of predictors in each logistic regression. All analyses were performed in the statistical software R v4.2.1 (R Core Team 2023), using an alpha level of 0.05 for significance testing.

### 2.7 Diet metric calculations

To compare the information gained before and after integration of DNA analysis, frequency occurrence was calculated using identifications based on visual‐only methods, then recalculated using identifications based on a combination of DNA‐based and visual methods. Frequency occurrence is a standard diet metric defined as the number of predator stomachs containing the prey taxon of interest divided by the total number of predator stomachs sampled. We focused on frequency occurrence because it is a robust and easily interpretable metric to describe composition of fish diets (Baker et al., 2014). The frequency occurrence of each clupeid prey taxon was calculated for all predator species combined and each predator species separately using diet data obtained from the portion of the survey that overlapped with the DNA analyses (2017–2022). Prior to calculating frequency occurrence with DNA‐supported data, prey identifications were updated using DNA results if barcoding was successful, whereas prey with unsuccessful barcoding results were assigned their previously given visual identification. *Brevoortia* sp. that were identified by DNA to genus level were combined with species‐level *Brevoortia tyrannus* identifications as one prey category (*Brevoortia* sp.) for calculations of frequency occurrence.

## 3 RESULTS

### 3.1 Visual‐based prey identifications

**FIGURE 2:**
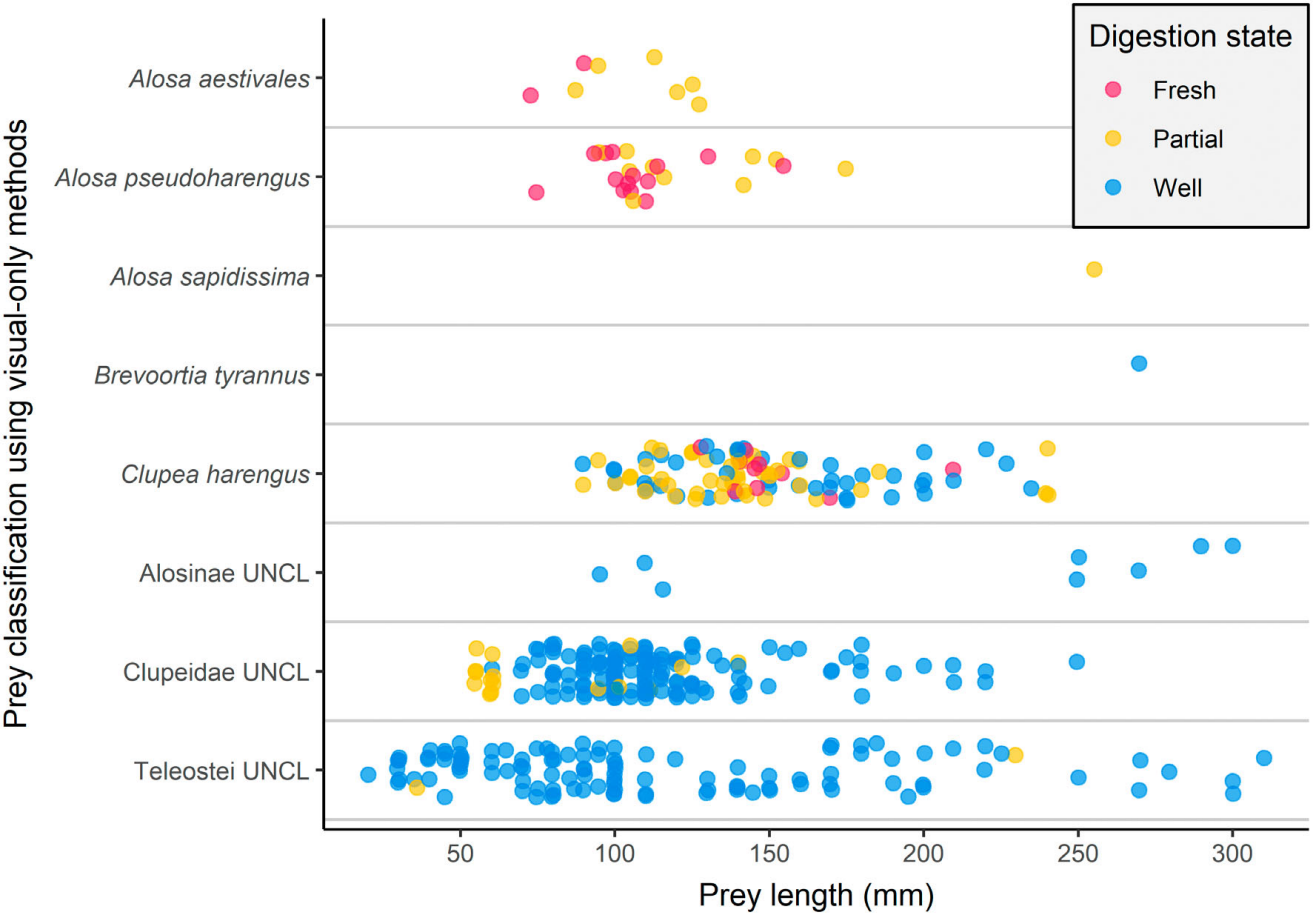
Classification using visual‐only methods of measurable (i.e. intact) piscine prey items observed in groundfish stomachs collected in the nearshore Gulf of Maine, 2012–2022. Each point represents an individual prey item and is coloured according to its digestion state (fresh, partial or well‐digested). The total length of each prey item is represented by its position along the horizontal axis. Random variation along the vertical axis within prey classes and 50% transparency were added to each point for visualization purposes only. The taxa displayed include species or lower‐resolution categories belonging to the Family Clupeidae as well as unclassified teleosts (i.e. bony fish remains). UNCL, unclassified.

Visual stomach content analysis provided species‐ or genus‐level identifications for 970 of 1797 (54.0%) piscine prey items observed in groundfish stomachs in the 2012–2022 surveys and 22 of the 179 (12.3%) samples selected for DNA analysis. Body size measurement was possible for 1202 of 1797 (66.9%) piscine prey items. Clupeid prey were visually assigned a family‐level identification for 183 observations and 144 observations were visually assigned a species‐level identification. Species‐ or genus‐level identification of piscine prey using visual methods was significantly less likely with smaller (*β*_SIZE_ = 0.007, standard error [SE] = 0.001; *X* ^2^[1, *N* = 1202] = 28.6, *p* < 0.001) and more digested specimens (*β*_WELL_ = −2.339, SE = 0.178; *X* ^2^[1, *N* = 1202] = 172.5, p < 0.001). Visually identifying clupeid prey items to species was seldom feasible for specimens <100 mm in length, especially partially or well‐digested specimens (Figure 2).

### 3.2 DNA barcoding of prey specimens

A database match was found for 122 of 179 (68.2%) specimens subjected to DNA analysis, with 109 identifications to species level and 13 to genus level (Tables 1 and 2). Twenty‐three specimens were assigned to *Alosa* spp. species (18 *A. pseudoharengus* and five *A. aestivalis*) using DNA analysis, of which 14 were previously assigned a lower resolution identification (Alosinae, Clupeidae or unclassified Teleostei) using visual methods (Table 2). Seventy‐seven specimens were assigned to *C. harengus*, of which 62 were visually assigned to family level (Table 2). Eight unclassified Teleostei were assigned to a clupeid species (Table 2), and two others were identified as non‐clupeid taxa, *M. bilinearis* and Atlantic saury *Scomberesox saurus* (Walbaum 1792). DNA‐based identification of trawl‐caught *A. pseudoharengus*, *A. aestivalis* and *A. sapidissima* (i.e. positive control samples) each matched their field identifications; perfect matches were obtained for each of the two *A. pseudoharengus* and two *A. aestivalis* field samples, and a 99.3% match was obtained for the one *A. sapidissima* (see Table S1 for amplicon sequences and BOLD sequence match numbers).

**TABLE 1:** Number of clupeid prey specimens successfully identified by DNA analysis from the stomach contents of five groundfish species caught in the nearshore Gulf of Maine, 2017–2022.

Common name	Scientific name	Silver hake (*Merluccius bilinearis*)	Spiny dogfish (*Squalus acanthias*)	Monkfish (*Lophius americanus*)	White hake (*Urophycis tenuis*)	Red hake (*Urophycis chuss*)
Alewife	*Alosa pseudoharengus*	15	1	1	–	1
Blueback herring	*Alosa aestivales*	5	–	–	–	–
Atlantic menhaden	*Brevoortia tyrannus*	–	6	–	1	–
Menhaden	*Brevoortia* sp.	1	8	3	1	–
Atlantic herring	*Clupea harengus*	67	4	2	3	1
Number of prey specimens analysed		123	29	16	8	3
(Number unsuccessful)		(33)	(10)	(10)	(3)	(1)

*Note*: The bottom rows indicate the total number of prey specimens analysed and the number of prey items that failed to provide an identification by predator species.

**TABLE 2:** Number of piscine prey specimens subjected to DNA analysis by their visual‐based identification (assigned prior to DNA analysis) and the resulting DNA‐based identifications.

Visual‐based ID		DNA‐based ID					
ID	*n*	Alewife	Blueback herring	Atlantic menhaden	Menhaden	Atlantic herring	Failed
Alewife	8	7	–	–	–	–	1 (13%)
Blueback herring	6	–	2	–	–	3	1 (17%)
Atlantic herring	8	–	–	–	–	6	2 (20%)
Alosinae UNCL	21	3	–	5	6	–	7 (33%)
Clupeidae UNCL	106	7	3	1	7	62	26 (25%)
Teleostei UNCL	30	1	–	1	–	6	20 (67%)
Measurements							
Number measureable		16	5	1	4	67	35
Size range (mm)		75–116	100–125	250	100–270	60–220	70–300
Mean size (mm)		99	110	250	200	105	127

*Note*: The ‘Failed’ column indicates the number (and percentage) of specimens that failed to provide an identification by DNA analysis. The bottom rows indicate the number of specimens that were measureable (i.e. intact) and the range and mean of size measurements (total length) by DNA‐based identification.

Of the 179 specimens, 157 were visually identified to higher than species levels, and DNA analysis was able to enhance this by assigning species to 104 of the specimens (104/157, 68.2%). In the remaining 22 cases in which specimens were previously (visually) assigned a species‐level identification, 15 were assigned the same species using DNA data, four did not have a taxonomy assignment (i.e. barcoding was unsuccessful) and the remaining three were assigned a conflicting identification: each involving a visually identified *A. aestivalis* that was identified by DNA as *C. harengus*. Sequences of clupeid prey species generated in this study and their perfect match in the BOLD are provided in Table S1.

The 13 genus‐level assignments were molecularly identified as either *Brevoortia tyrannus* or *Brevoortia patronus*. This was because these query sequences matched to both *B*. *tyrannus* and *B*. *patronus* voucher sequences, contributed by different researchers in GenBank. While keeping both species as possibilities, we think *B*. *tyrannus* is more likely to be the correct identification considering the geographical range of our specimens.

**FIGURE 3:**
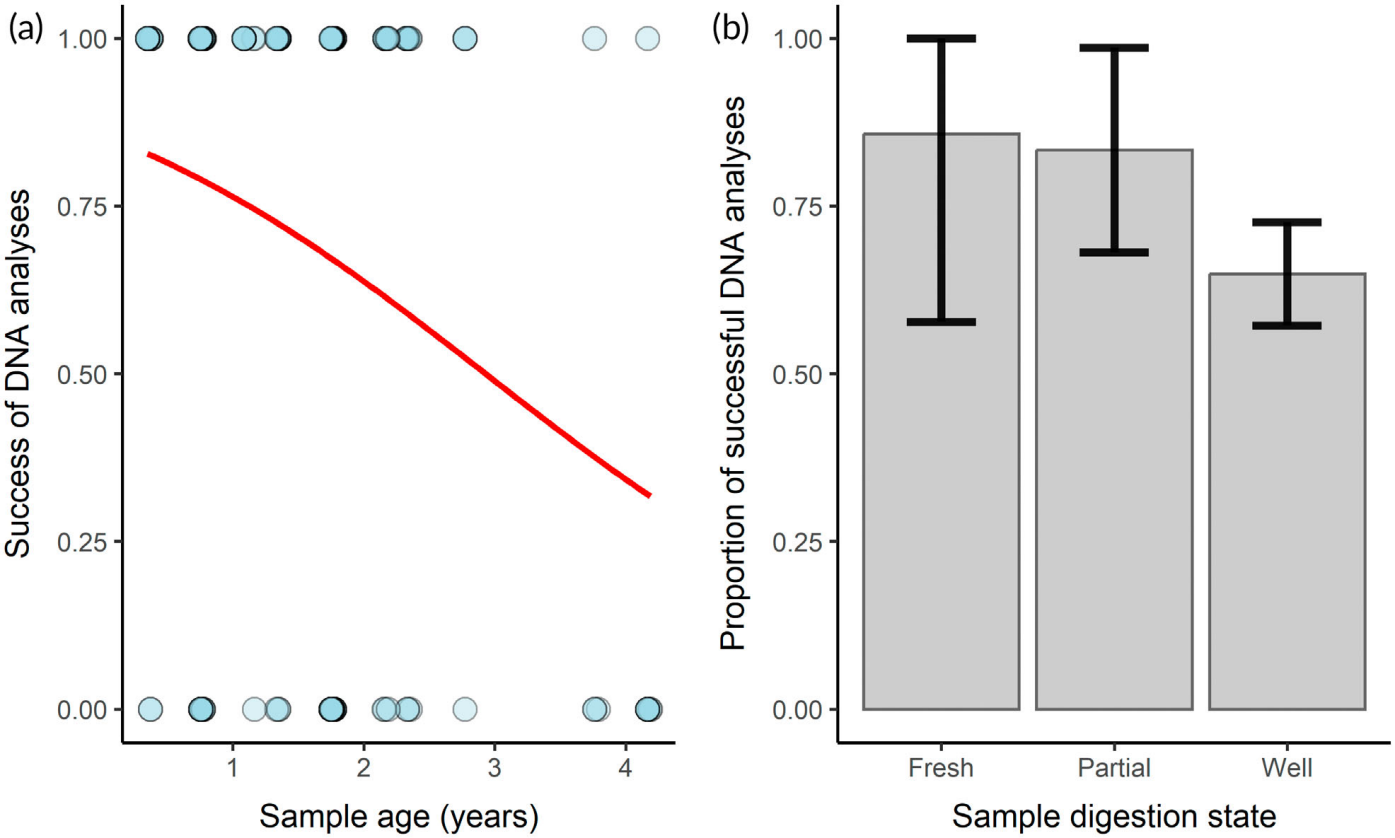
Relationships between the success of DNA analysis in identifying potential *Alosa* sp. prey specimens (*n* = 179) and the sample age (a) and digestion state (b). Blue points in (a) represent individual prey specimens, and 50% transparency was added to each point to aid in visualization of overlapping points. One and zero along the vertical axis indicate success and failure, respectively, of DNA‐based identification for each specimen. Sample age was defined as the time in years between initial preservation of the groundfish stomach in ethanol and the DNA extraction. The red line represents the predicted probability of success based on a logistic regression with sample age as the only predictor. Error bars in (b) represent 95% confidence intervals. By digestion state, DNA analysis was performed on seven fresh, 24 partial and 148 well‐digested specimens.

The success of DNA analysis in providing a database match for prey items was negatively related to sample age (*β* = −0.559, SE = 0.193; *X* ^2^[1, *N* = 179] = 8.4, *p* = 0.004; Figure 3a) but not significantly related to digestion state (*X* ^2^[2, *N* = 179] = 2.2, *p* = 0.33; Figure 3b). The interaction term in the initial model was not significant (*X* ^2^[2, *N* = 179] = 5.062, *p* = 0.08). Specimens that were visually assigned to the unclassified Teleostei category had low rates (33.3%) of success in DNA analysis, whereas most unclassified Alosinae (66.6%) and Clupeidae (75.5%) were successfully identified to species (Table 2). Overall, 55 specimens failed to have any taxonomy assignment based on DNA analysis. In these cases, PCR failed to amplify any products (*n* = 53) or amplified non‐specific products that did not match to any known reference sequences in any of the three sequence databases.

### 3.3 Diet metric calculations

**FIGURE 4:**
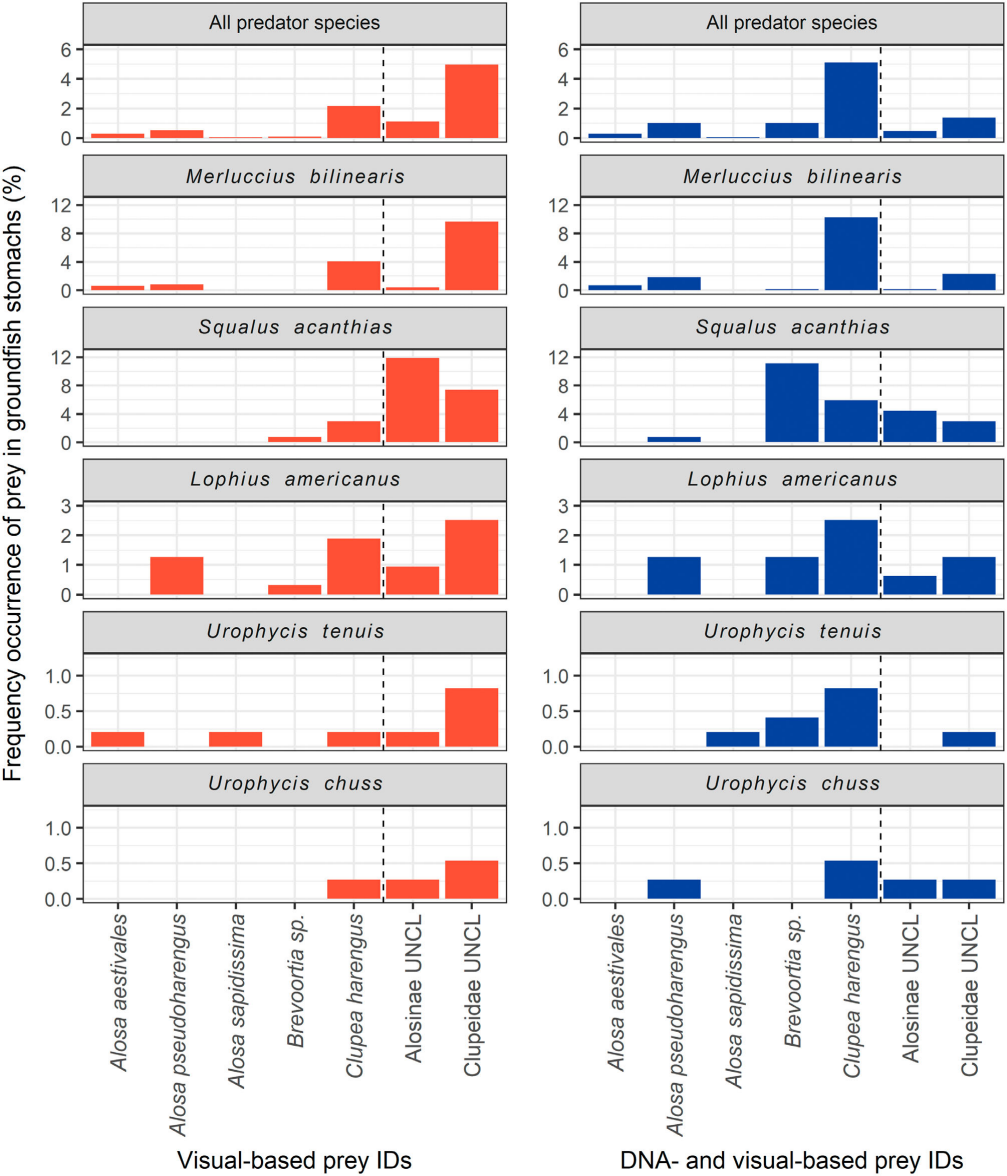
Calculated frequency occurrence values of clupeid prey taxa in stomachs of *Merluccius bilinearis* (*n* = 868), *Squalus acanthias* (*n* = 135), *Lophius americanus* (*n* = 318), *Urophycis tenuis* (*n* = 487) and *Urophycis chuss* (*n* = 371) collected in the nearshore Gulf of Maine, 2017–2022. The left column displays values based on visual‐only methods for identification. The right column displays values based on the combination of DNA‐ and visual‐based identification of prey. Vertical dashed lines separate prey categories that are species or genus level (left of dashed line) and lower resolution classifications (right of dashed line). Sample sizes of prey specimens subjected to DNA analysis by predator species are provided in Table 1. UNCL, unclassified.

Integrating visual‐ and DNA‐based prey identifications improved diet metric calculations by reducing frequency occurrence (FO) values in lower‐resolution categories, increasing values in species‐ or genus‐level classifications, and in few cases changing zero to non‐zero values or vice versa (Figure 4). In calculations for all predator species combined, the FO of unclassified Clupeidae was reduced from 4.96% in visual‐only analysis to 1.38% using a combination of DNA and visual methods; the FO of unclassified Alosinae also reduced from 1.10% to 0.64%. Within predator species, the integration of DNA‐based identifications resulted in increased FO of *C. harengus* in each predator species (e.g. from 4.03% to 10.25% in *M. bilinearis*), increased FO of *Brevoortia* sp. from 0.74% to 11.11% in *S. acanthias* and 0.31% to 1.26% in *L. americanus*, and increased FO of *A. pseudoharengus* from 0.81% to 1.84% in *M. bilinearis*. The integration of DNA results also converted zero to non‐zero FO values, specifically for *A. pseudoharengus* prey in *S. acanthias* and *U. chuss*. A non‐zero FO of *A. aestivalis* in *U. tenuis* was changed to zero due to one specimen receiving a DNA‐based identification that conflicted with visual identification. Despite two similar conflicting results, the FO of *A. aestivalis* in *M. bilinearis* increased from 0.58% to 0.68% following integration of DNA results.

## 4 DISCUSSION

DNA‐based identification enhanced the resolution of anadromous (*Alosa* spp.) and marine clupeids (*C. harengus*, *Brevoortia* sp.) in diets of marine groundfishes compared to visual stomach content analysis. Interspecific morphological similarities and digestive degradation of distinguishing characteristics (ventral scutes, peritoneum pigmentation, body shape) contributed to a higher number of family‐level (183) than species‐level (144) identifications among clupeids in the visual analysis. Taxonomic resolution of prey identification varies considerably among stomach content studies, but low‐to‐moderate rates (i.e. ~10%–60%) of species‐level identification are common for piscine prey (e.g. Aguilar et al., 2017; Buckland et al., 2017; Smith & Link, 2010). Generally, the resolution of prey identification in stomach content analysis can depend on various factors, including prey condition (Buckland et al., 2017), predator feeding mode and prey handling (Scharf et al., 1997), and the life stages of prey involved (Carreon‐Martinez et al., 2011). In our study, consumed clupeids ~100 mm or shorter in length were rarely identified to species using visual analysis but were reliably identified to species using DNA barcoding. Overall, DNA barcoding showed moderate‐to‐high success (68.2%) in providing species‐ or genus‐level identifications of clupeid prey, including a >60% identification rate for samples categorized as ‘well‐digested’. This is comparable to success rates reported in previous studies that applied DNA barcoding for identification of piscine prey in stomach contents (~60%–90%; Paine et al., 2007, Dunn et al., 2010, Valdez‐Moreno et al., 2012, Paquin et al., 2014, Moran et al., 2016, Aguilar et al., 2017). The 5‐fold increase (from 12.3% to 68.2%) in species‐ or genus‐level identification rate of the 179 specimens subjected to DNA analysis represents a substantial gain in trophic information pertaining to interactions among ecologically diverse clupeids and marine predators.

Integrating the higher‐resolution prey identifications provided by DNA analysis with the visual stomach content analysis enhanced precision and accuracy of the frequency occurrence diet metric in our study. As a result of 104 improvements to taxonomic resolution, frequency occurrence values were redistributed from lower‐resolution to higher‐resolution prey classifications. Accuracy of frequency occurrence was improved through the correction of three species identifications, each with *C. harengus* being identified visually as *A. aestivalis* due to similarities of ventral scutes among smaller individuals (Able & Fahay, 1998; Fischbach et al., 2023). While we evaluated changes only in frequency occurrence here (see Falke et al., 2024 for a detailed diet analysis), similar effects of improved prey identification would be expected across other commonly used dietary metrics, including numerical, mass‐ and volume‐based estimates as well as metrics incorporating prey availability (Baker et al., 2014). Evaluating how molecular identification influences these additional metrics would provide a stronger basis for comparing the impacts of DNA‐based methods in diet assessments. Improving the precision and accuracy of these diet metrics is ultimately valuable for the parameterization and validation of realistic food‐web and ecosystem models (Livingston, 1985; Metcalf et al., 2008; Pethybridge et al., 2018). Combining morphological and molecular approaches might be the most effective approach for obtaining robust diet datasets and ecological insights because it can be used selectively to balance resource requirements (i.e. time, monetary cost, expertise) with the depth of information gained from different methods (Aguilar et al., 2017; Amundsen & Sánchez‐Hernández, 2019; Berry et al., 2015; Casper et al., 2007; Oehm et al., 2017; Tollit et al., 2009; Traugott et al., 2021). For instance, Oehm et al. (2017) recommended the use of pellets (a morphological analysis) in combination with molecular prey identification to study piscivorous bird diets because pellets provided comprehensive trophic information with low sampling effort, while DNA analysis provided the best detectability of prey species. Additional information would have been gained by initiating DNA analysis earlier in our study (i.e. prior to 2017), targeting other prey families or sequencing additional samples categorized as ‘unidentifiable fish’. Nonetheless, the 3‐fold reduction in frequency occurrence values attributed to unclassified clupeids (all groundfish species combined from 2017 to 2022) represents a meaningful improvement in diet metric quality following the integration of DNA analysis.

The improvements to diet information help clarify the role of anadromous prey fishes in marine food webs. While our DNA barcoding effort focused on potential *Alosa* spp. prey, only 23 of 122 successfully matched specimens were identified as *Alosa* spp. Most unclassified Clupeidae and unclassified Alosinae specimens were identified via barcoding as *C. harengus* and *Brevoortia* sp., respectively. The largest changes in frequency occurrence values among genus‐ or species‐level classifications were therefore observed for *C. harengus* (e.g. in *M. bilinearis*) and *Brevoortia* sp. (e.g. in *S. acanthias*). However, DNA analysis provided evidence of *Alosa* spp. predation in *S. acanthias* and *U. chuss*, which was not obtained through the visual analysis. These DNA‐confirmed occurrences were evaluated in the context of a much larger stomach content dataset from Falke et al. (2024), which included >2000 stomachs overlapping the DNA barcoding effort from 2017 to 2022. Across this broader dataset, frequency occurrence for *Alosa* spp. remained low (<2%) for all predator species, even after incorporating the improved taxonomic resolution provided by DNA barcoding. These results are therefore consistent with recent studies that report low contemporary contributions of *Alosa* spp. in groundfish diets (Falke et al., 2024; McDermott et al., 2015; Smith & Link, 2010; Willis et al., 2017). The low contemporary diet contributions of *Alosa* spp. are probably related to their population decline, whereas historic levels of *Alosa* spp. abundance were hypothesized drivers of groundfish productivity in the Gulf of Maine (Ames, 2004; Ames & Lichter, 2013). Offshore sampling of fish stomach contents on the northeast US continental shelf has been conducted for several decades (Link & Almeida, 2000) and the percentage composition of *Alosa* spp. in groundfish stomachs is generally negligible (i.e. 0%–3%; Garrison & Link, 2000, Smith & Link, 2010). However, the resolution of piscine prey in these studies is often coarse, with upward of 15% diet composition attributed to unclassified clupeids and larger amounts attributed to ‘unidentified fish’. McDermott et al. (2015) found that frequencies of *Alosa* spp. and groundfish interactions are significantly higher in near‐coastal waters than offshore sampling areas, while Falke et al. (2024) suggested that low *Alosa* spp. contributions in groundfish diets in the nearshore Gulf of Maine reflect their low availability compared to more available piscine prey, primarily *C. harengus* and *M. bilinearis*. Thus, although estuarine biomass of *Alosa* spp. has increased alongside recent restoration efforts in Maine (Stevens et al., 2024), their persistently low frequency occurrence in groundfish diets since inshore stomach sampling began in 2012 does not clearly reflect ecosystem‐level responses extending to marine predator diets (Falke et al., 2024). In contrast, a recent stomach content analysis of Atlantic bluefin tuna *Thunnus thynnus* L. in the Gulf of Maine provided some evidence of increasing diet prevalence of *Alosa* spp. relative to previous studies (Nadeau et al., 2025). Integrating targeted DNA analyses could resolve anadromous versus marine clupeid diet contributions with greater precision and thereby improve the sensitivity of diet monitoring needed to detect future ecosystem effects as *Alosa* spp. populations continue to respond to restoration.

The ability to measure sizes of consumed prey along with high‐resolution identification provides additional ecological insights, including interactions between groundfish and *Alosa* spp. life stages, and is another major benefit of combining morphological and molecular approaches for prey identification (Garrido et al., 2024). Lengths (TL) of consumed *A. pseudoharengus* and *A. aestivalis* identified using DNA ranged from 75 to 116 and 100 to 125 mm, respectively. Based on otolith aging of *A. pseudoharengus* and *A. aestivalis* (Stevens et al., 2021), these sizes most likely correspond to age‐0 or age‐1 individuals that have recently out‐migrated from freshwater, rather than adults returning to freshwater to spawn. In theory, the consumption of *Alosa* spp. out‐migrating from freshwater conveys a cross‐ecosystem transfer of nutrients between freshwater and marine systems (Barber et al., 2018; Smith et al., 2016), whereas consumption of adult *Alosa* spp. or marine clupeids conveys transfer of nutrients predominantly derived within the marine ecosystem. Visually distinguishing anadromous and marine clupeids in stomach contents is often not possible, especially when specimens are juvenile life stages. Visual identification of larval or juvenile fishes in stomach contents can be limited to the first 2 h of digestion time (Legler et al., 2010; Schooley et al., 2008). The influence of seasonality in *Alosa* spp. life history (e.g. juvenile out‐migration) on nearshore trophic dynamics could therefore be obscured in diet information derived from visual methods. DNA‐based prey identification provides a more precise tool for resolving trophic interactions involving juvenile clupeids, making it valuable for understanding the ecosystem connections that *Alosa* spp. form along the diadromous watersheds‐to‐ocean continuum and their potential responses to ecosystem changes (Hare et al., 2021; Ouellet et al., 2022).

Maximizing the effectiveness of molecular approaches to prey identification (e.g. barcoding success rates in our study) is key to establishing its use in diet studies. Consistent with known limitations of DNA‐based diet analyses, barcoding success in our study reflected a combination of sample preservation conditions, digestion state and reference database quality. Our barcoding success declined significantly with sample age and to a lesser extent with prey digested state. The observed decline in barcoding success with increasing sample age suggests a possible issue with the long‐term preservation of samples in ethanol and potential degradation of DNA over time. Storing samples in ethanol is a valid preservation technique for DNA analysis (Weber & Lundgren, 2009), but we recommend carefully considering the proper ethanol quality (e.g. ≥95% concentration, minimal dilution from residual water or digestive fluids) and quantity (i.e. sufficient ethanol volume to fully submerge samples, maintaining a high ethanol‐to‐tissue ratio during storage). To have a higher probability of barcoding success, freezing samples or extracting DNA promptly after collection have been suggested as better alternatives to continued preservation in ethanol (Aguilar et al., 2017; Valdez‐Moreno et al., 2012), but these methods are not always practicable. Previous studies report mixed results regarding the influence of digestion state on barcoding success (Aguilar et al., 2017; Berry et al., 2015; Moran et al., 2016; Paine et al., 2007; Paquin et al., 2014; Valdez‐Moreno et al., 2012). DNA identification of extremely degraded samples (e.g. chyme, particulates, bones) is generally far less likely (Aguilar et al., 2017; Carreon‐Martinez et al., 2011), but such states of degradation were not included in our sample selection. The success rate for samples classified as ‘well‐digested’ was still moderate to high (65%) but lower than ‘fresh’ and ‘partial’ samples (>83%), and this might have been statistically significant with larger sample sizes of the latter categories. Barcoding success rates can also depend on several other factors, such as sequencing error (e.g. incorrect base calls), contamination (e.g. extraction of non‐target DNA), diagnostic gene selection or reference database quality (Antil et al., 2023; Berry et al., 2015; King et al., 2008; Leray et al., 2013; Sakaguchi et al., 2017). For example, the inability to resolve *Brevoortia* sp. to species in our study might be due to errors in sequence identifications in reference databases. In 13 cases, BOLD sequence comparisons yielded matches to two *Brevoortia* spp. (*B. tyrannus* and *B. patronus*), and all follow‐up comparisons to Smithsonian's voucher specimens in GenBank (Accession number: OP057013) indicated the highest percentage match with *B. patronus*. However, *B. tyrannus* is the only species in the *Brevoortia* genus whose range is known to overlap with our study area. While diet information was substantially enhanced by incorporating molecular evidence, further improvements will depend on continued refinement of reference data.

Our study highlights the potential for DNA‐based prey identification to enhance our understanding of ecosystem processes by improving the quality of trophic information, and it identifies future directions for monitoring the role of anadromous fishes as prey in marine food webs. The targeted integration of DNA analysis with visual prey identification effectively clarified the taxonomic resolution of anadromous and marine clupeids in marine groundfish stomach contents, resulting in more precise and accurate quantification of their relative diet contributions. Combining targeted molecular methods with conventional diet assessment methods such as stomach content, fatty acid or stable isotope analyses might be the best approach for obtaining high‐quality empirical data on food web interactions while balancing research costs and project objectives. DNA‐based diet assessments are therefore a promising approach for improving the empirical robustness of ecosystem models that support fisheries management (Pethybridge et al., 2018; Traugott et al. 2020). DNA‐based methods add taxonomic resolution to diet analyses and are most informative when applied alongside established visual approaches, particularly when specific ecological or management questions require finer taxonomic resolution. Resource requirements for DNA‐based diet analyses can vary widely depending on study design, sequencing approach and laboratory infrastructure; however, the ongoing expansion of genomic services and declining sequencing costs are increasing their accessibility. Accordingly, the decision to incorporate molecular tools should be guided by study objectives and resource constraints. The improvements to diet information shown in our study are particularly useful considering the diverse ecological roles (e.g. ontogenetic habitat use, nutrient transfer) of clupeid species and life stages between species. For instance, individual‐based ecosystem models incorporate traits such as life history to characterize food web dynamics, including nutrient and energy exchanges across ecosystems (DeAngelis & Gross, 1992; Grimm & Railsback, 2005). Broadening DNA‐based identification of anadromous prey will provide better understanding of their trophic ecology, including their role as prey in other marine predators (e.g. raptors, pinnipeds, cetaceans). This could also be useful in monitoring fisheries responses to ecosystem changes, such as freshwater restoration efforts that could increase the anadromous prey base (Hall et al., 2012) or rapid changes in temperature regimes along the US northeast continental shelf that could alter species distributions (Friedland et al., 2025; Mills et al., 2024). Improving resources and strategies for DNA analyses through optimizing sampling effort, preservation, sequencing technology and reference databases will enhance its use in diet studies.

## AUTHOR CONTRIBUTIONS

All authors conceptualized ideas for the manuscript. S.R. performed all laboratory processing of stomach contents and DNA extractions, Y.L. interpreted DNA sequencing results, L.P.F. led data analyses and manuscript preparation, and all authors reviewed and edited the manuscript.

## Supporting information


**FIGURE S1.** Pictures of *Alosa pseudoharengus*, *Alosa aestivalis* and *Clupea harengus* specimens representing the three digestion states (fresh, partial and well). Except for the fresh *C. harengus*, which is wild‐caught, all specimens were sampled from groundfish stomachs in the nearshore Gulf of Maine and positively identified to species using DNA barcoding in the present study.
**TABLE S1.** Sequences of clupeid species sampled in the nearshore Gulf of Maine and their matching sequence record in the Barcode of Life Data System (BOLD).

## References

[jfb70415-bib-0001] Able, K. W. , & Fahay, M. P. (1998). The first year in the life of estuarine fishes in the middle Atlantic bight. Rutgers University Press.

[jfb70415-bib-0002] Acolas, M.‐L. , & Lambert, P. (2016). Life histories of anadromous fishes. In P. Morais & F. Daverat (Eds.), An introduction to fish migration (pp. 55–77). CRC Press.

[jfb70415-bib-0003] Aguilar, R. , Ogburn, M. B. , Driskell, A. C. , Weigt, L. A. , Groves, M. C. , & Hines, A. H. (2017). Gutsy genetics: Identification of digested piscine prey items in the stomach contents of sympatric native and introduced warmwater catfishes via DNA barcoding. Environ Biology of Fishes, 100, 325–336.

[jfb70415-bib-0004] Ames, E. P. , & Lichter, J. (2013). Gadids and alewives: Structure within complexity in the Gulf of Maine. Fisheries Research, 141, 70–78.

[jfb70415-bib-0005] Ames, E. T. (2004). Atlantic cod stock structure in the Gulf of Maine. Fisheries, 29, 10–28.

[jfb70415-bib-0006] Amundsen, P.‐A. , & Sánchez‐Hernández, J. (2019). Feeding studies take guts – Critical review and recommendations of methods for stomach contents analysis in fish. Journal of Fish Biology, 95, 1364–1373. 31589769 10.1111/jfb.14151

[jfb70415-bib-0007] Antil, S. , Abraham, J. S. , Sripoorna, S. , Maurya, S. , Dagar, J. , Makhija, S. , Bhagat, P. , Gupta, R. , Sood, U. , Lal, R. , & Toteja, R. (2023). DNA barcoding, an effective tool for species identification: A review. Molecular Biology Reports, 50, 761–775. 36308581 10.1007/s11033-022-08015-7

[jfb70415-bib-0008] Baker, R. , Buckland, A. , & Sheaves, M. (2014). Fish gut content analysis: Robust measures of diet composition. Fish and Fisheries, 15, 170–177.

[jfb70415-bib-0009] Barber, B. L. , Gibson, A. J. , O'Malley, A. J. , & Zydlewski, J. (2018). Does what goes up also come down? Using a recruitment model to balance alewife nutrient import and export. Marine and Coastal Fisheries, 10, 236–254.

[jfb70415-bib-0010] Benson, D. A. , Karsch‐Mizrachi, I. , Lipman, D. J. , Ostell, J. , & Wheeler, D. L. (2004). GenBank: update. Nucleic Acids Research, 32, D23–D26. 14681350 10.1093/nar/gkh045 PMC308779

[jfb70415-bib-0011] Berry, O. , Bulman, C. , Bunce, M. , Coghlan, M. , Murray, D. C. , & Ward, R. D. (2015). Comparison of morphological and DNA metabarcoding analyses of diets in exploited marine fishes. Marine Ecology Progress Series, 540, 167–181.

[jfb70415-bib-0012] Buckland, A. , Baker, R. , Loneragan, N. , & Sheaves, M. (2017). Standardising fish stomach content analysis: The importance of prey condition. Fisheries Research, 196, 126–140.

[jfb70415-bib-0013] Carreon‐Martinez, L. , Johnson, T. B. , Ludsin, S. A. , & Heath, D. D. (2011). Utilization of stomach content DNA to determine diet diversity in piscivorous fishes. Journal of Fish Biology, 78, 1170–1182. 21463313 10.1111/j.1095-8649.2011.02925.x

[jfb70415-bib-0014] Casper, R. M. , Jarman, S. N. , Deagle, B. E. , Gales, N. J. , & Hindell, M. A. (2007). Detecting prey from DNA in predator scats: A comparison with morphological analysis, using *Arctocephalus* seals fed a known diet. Journal of Experimental Marine Biology and Ecology, 347, 144–154.

[jfb70415-bib-0015] da Silveira, E. L. , Semmar, N. , Cartes, J. E. , Tuset, V. M. , Lombarte, A. , Ballester, E. L. , & Vaz‐dos‐Santos, A. M. (2020). Methods for trophic ecology assessment in fishes: A critical review of stomach analyses. Reviews in Fisheries Science & Aquaculture, 28, 71–106.

[jfb70415-bib-0016] Deagle, B. E. , Gales, N. J. , Evans, K. , Jarman, S. N. , Robinson, S. , Trebilco, R. , & Hindell, M. A. (2007). Studying seabird diet through genetic analysis of faeces: A case study on macaroni penguins (Eudyptes chrysolophus). PLoS One, 2, e831. 17786203 10.1371/journal.pone.0000831 PMC1959119

[jfb70415-bib-0017] DeAngelis, D. L. , & Gross, L. J. (1992). Individual‐based models and approaches in ecology. Chapman & Hall.

[jfb70415-bib-0018] Dunn, M. R. , Szabo, A. , McVeagh, M. S. , & Smith, P. J. (2010). The diet of deepwater sharks and the benefits of using DNA identification of prey. Deep Sea Research, Part I: Oceanographic Research Papers, 57, 923–930.

[jfb70415-bib-0019] Falke, L. P. , Smith, B. E. , Rowe, S. , Peters, R. J. , & Sheehan, T. F. (2024). Trophic ecology of groundfishes in nearshore areas of the Gulf of Maine. Journal of Fish Biology, 106, 1095–1111. 10.1111/jfb.16026 39648788 PMC12038783

[jfb70415-bib-0020] Fischbach, V. , Finke, A. , Moritz, T. , Polte, P. , & Thieme, P. (2023). A staging system for Atlantic herring (*Clupea harengus*) larvae based on external morphology and skeletal development. Limnology and Oceanography, Methods, 21, 357–376.

[jfb70415-bib-0021] Friedland, K. D. , Scopel, L. C. , Yang, X. , Gaichas, S. K. , & Rokosz, K. J. (2025). Species richness in the northeast US continental shelf ecosystem: Climate‐driven trends and perturbations. PLOS Climate, 4, e0000557.

[jfb70415-bib-0022] Garrido, S. , Albo‐Puigserver, M. , & Moyano, M. (2024). Larval trophic ecology of small pelagic fishes: A review of recent advances and pathways to fill remaining knowledge gaps. Marine Ecology Progress Series SPF2, 741, 127–143. 10.3354/meps14543

[jfb70415-bib-0023] Garrison, L. P. , & Link, J. S. (2000). Diets of five hake species in the northeast United States continental shelf ecosystem. Marine Ecology Progress Series, 204, 243–255.

[jfb70415-bib-0024] Grimm, V. , & Railsback, S. F. (2005). Individual‐based modeling and ecology. Princeton Univ. Press.

[jfb70415-bib-0025] Hajibabaei, M. , Janzen, D. H. , Burns, J. M. , Hallwachs, W. , & Hebert, P. D. N. (2006). DNA barcodes distinguish species of tropical Lepidoptera. Proceedings of the National Academy of Sciences, 103, 968–971. 10.1073/pnas.0510466103 PMC1327734 16418261

[jfb70415-bib-0026] Hall, C. J. , Jordaan, A. , & Frisk, M. G. (2011). The historic influence of dams on diadromous fish habitat with a focus on river herring and hydrologic longitudinal connectivity. Landscape Ecology, 26, 95–107.

[jfb70415-bib-0027] Hall, C. J. , Jordaan, A. , & Frisk, M. G. (2012). Centuries of anadromous forage fish loss: Consequences for ecosystem connectivity and productivity. Bioscience, 62, 723–731.

[jfb70415-bib-0028] Handy, S. M. , Deeds, J. R. , Ivanova, N. V. , Hebert, P. D. N. , Hanner, R. , Ormos, A. , Weigt, L. A. , Moore, M. M. , & Yancy, H. F. (2011). A single laboratory validated method for the generation of DNA barcodes for the identification of fish for regulatory compliance. Journal of AOAC International, 94(1), 201–210. 21391497

[jfb70415-bib-0029] Hare, J. A. , Borggaard, D. L. , Alexander, M. A. , Bailey, M. M. , Bowden, A. A. , Damon‐Randall, K. , Didden, J. T. , Hasselman, D. J. , Kerns, T. , McCrary, R. , McDermott, S. , Nye, J. A. , Pierce, J. , Schultz, E. T. , Scott, J. D. , Starks, C. , Sullivan, K. , & Beth Tooley, M. (2021). A review of river herring science in support of species conservation and ecosystem restoration. Marine and Coastal Fisheries, 13, 627–664.

[jfb70415-bib-0030] Hebert, P. D. N. , Cywinska, A. , Ball, S. L. , & deWaard, J. R. (2003). Biological identifications through DNA barcodes. Proceedings of the Royal Society of London. Series B, Biological Sciences, 270, 313–321. 10.1098/rspb.2002.2218 PMC1691236 12614582

[jfb70415-bib-0031] Hynes, H. B. N. (1950). The food of fresh‐water sticklebacks (*Gasterosteus aculeatus* and *Pygosteus pungitius*), with a review of methods used in studies of the food of fishes. Journal of Animal Ecology, 19, 36–58.

[jfb70415-bib-0032] Hyslop, E. J. (1980). Stomach contents analysis: A review of methods and their application. Journal of Fish Biology, 17, 411–429.

[jfb70415-bib-0033] Jarman, S. N. , Gales, N. J. , Tierney, M. , Gill, P. C. , & Elliott, N. G. (2002). A DNA‐based method for identification of krill species and its application to analysing the diet of marine vertebrate predators. Molecular Ecology, 11, 2679–2690. 12453250 10.1046/j.1365-294x.2002.01641.x

[jfb70415-bib-0034] Jones, A. W. , Dalton, C. M. , Stowe, E. S. , & Post, D. M. (2010). Contribution of declining anadromous fishes to the reproductive investment of a common piscivorous seabird, the double‐crested cormorant (*Phalacrocorax auritus*). The Auk, 127, 696–703.

[jfb70415-bib-0035] Juanes, F. , Marks, R. E. , McKown, K. A. , & Conover, D. O. (1993). Predation by age‐0 bluefish on age‐0 anadromous fishes in the Hudson River estuary. Transactions of the American Fisheries Society, 122, 348–356.

[jfb70415-bib-0036] King, R. A. , Read, D. S. , Traugott, M. , & Symondson, W. O. C. (2008). INVITED REVIEW: Molecular analysis of predation: A review of best practice for DNA‐based approaches. Molecular Ecology, 17, 947–963. 18208490 10.1111/j.1365-294X.2007.03613.x

[jfb70415-bib-0037] Leach, L. , Stevens, J. R. , & Cammen, K. (2024). Pinniped response to diadromous fish restoration in the Penobscot River estuary. Frontiers in Conservation Science, 5, 1408982. 10.3389/fcosc.2024.1408982

[jfb70415-bib-0038] Legler, N. D. , Johnson, T. B. , Heath, D. D. , & Ludsin, S. A. (2010). Water temperature and prey size effects on the rate of digestion of larval and early juvenile fish. Transactions of the American Fisheries Society, 139, 868–875.

[jfb70415-bib-0039] Leray, M. , Yang, J. Y. , Meyer, C. P. , Mills, S. C. , Agudelo, N. , Ranwez, V. , Boehm, J. T. , & Machida, R. J. (2013). A new versatile primer set targeting a short fragment of the mitochondrial COI region for metabarcoding metazoan diversity: Application for characterizing coral reef fish gut contents. Frontiers in Zoology, 10, 34. 23767809 10.1186/1742-9994-10-34 PMC3686579

[jfb70415-bib-0040] Limburg, K. E. , & Waldman, J. R. (2009). Dramatic declines in North Atlantic diadromous fishes. Bioscience, 59, 955–965.

[jfb70415-bib-0041] Link, J. S. , & Almeida, F. P. (2000). An overview and history of the food web dynamics program of the Northeast Fisheries Science Center, Woods Hole, Massachusetts. NOAA Technical Memorandum NMFS‐NE‐159.

[jfb70415-bib-0042] Livingston, P. A. (1985). An ecosystem model evaluation: The importance of fish food habits data. Marine Fisheries Review, 47, 9–12.

[jfb70415-bib-0043] McDermott, S. P. , Bransome, N. C. , Sutton, S. E. , Smith, B. E. , Link, J. S. , & Miller, T. J. (2015). Quantifying alosine prey in the diets of marine piscivores in the Gulf of Maine. Journal of Fish Biology, 86, 1811–1829. 25943427 10.1111/jfb.12692

[jfb70415-bib-0044] MEDMR . (2023). Maine‐New Hampshire Inshore Trawl Survey. https://www.maine.gov/dmr/science/fisheries-monitoring-assessment/maine-new-hampshire-inshore-trawl-survey (last accessed 20 December 2023)

[jfb70415-bib-0045] Metcalf, S. J. , Dambacher, J. M. , Hobday, A. J. , & Lyle, J. M. (2008). Importance of trophic information, simplification and aggregation error in ecosystem models. Marine Ecology Progress Series, 360, 25–36.

[jfb70415-bib-0046] Mills, K. E. , Kemberling, A. , Kerr, L. A. , Lucey, S. M. , McBride, R. S. , Nye, J. A. , Pershing, A. J. , Barajas, M. , & Lovas, C. S. (2024). Multispecies population‐scale emergence of climate change signals in an ocean warming hotspot. ICES Journal of Marine Science, 81, 375–389.

[jfb70415-bib-0047] Moran, Z. , Orth, D. J. , Schmitt, J. D. , Hallerman, E. M. , & Aguilar, R. (2016). Effectiveness of DNA barcoding for identifying piscine prey items in stomach contents of piscivorous catfishes. Environ Biology of Fishes, 99, 161–167.

[jfb70415-bib-0048] Nadeau, S. B. , Logan, J. M. , Carlucci, J. , Zydleweski, G. , & Golet, W. J. (2025). Dietary shifts and energetics of Atlantic bluefin tuna *Thunnus thynnus* in the Gulf of Maine. Marine Ecology Progress Series, 759, 71–87.

[jfb70415-bib-0049] Nielsen, J. M. , Clare, E. L. , Hayden, B. , Brett, M. T. , & Kratina, P. (2018). Diet tracing in ecology: Method comparison and selection. Methods in Ecology and Evolution, 9, 278–291.

[jfb70415-bib-0050] Oehm, J. , Thalinger, B. , Eisenkölbl, S. , & Traugott, M. (2017). Diet analysis in piscivorous birds: What can the addition of molecular tools offer? Ecology and Evolution, 7, 1984–1995. 28331605 10.1002/ece3.2790 PMC5355203

[jfb70415-bib-0051] Ouellet, V. , Collins, M. J. , Kocik, J. F. , Saunders, R. , Sheehan, T. F. , Ogburn, M. B. , & Trinko Lake, T. (2022). The diadromous watersheds‐ocean continuum: Managing diadromous fish as a community for ecosystem resilience. Frontiers in Ecology and Evolution, 10, 1–29.

[jfb70415-bib-0052] Paine, M. A. , McDowell, J. R. , & Graves, J. E. (2007). Specific identification of Western Atlantic Ocean scombrids using mitochondrial DNA cytochrome c oxidase subunit I (COI) gene region sequences. Bulletin of Marine Science, 80, 353–367.

[jfb70415-bib-0053] Paquin, M. M. , Buckley, T. W. , Hibpshman, R. E. , & Canino, M. F. (2014). DNA‐based identification methods of prey fish from stomach contents of 12 species of eastern North Pacific groundfish. Deep Sea Research Part I: Oceanographic Research Papers, 85, 110–117.

[jfb70415-bib-0054] Pentinsaari, M. , Ratnasingham, S. , Miller, S. E. , & Hebert, P. D. N. (2020). BOLD and GenBank revisited – Do identification errors arise in the lab or in the sequence libraries? PLoS One, 15(4), e0231814. 32298363 10.1371/journal.pone.0231814 PMC7162515

[jfb70415-bib-0055] Pethybridge, H. R. , Choy, C. A. , Polovina, J. J. , & Fulton, E. A. (2018). Improving marine ecosystem models with biochemical tracers. Annual Review of Marine Science, 10, 199–228. 10.1146/annurev-marine-121916-063256 29298140

[jfb70415-bib-0056] Sakaguchi, S. O. , Shimamura, S. , Shimizu, Y. , Ogawa, G. , Yamada, Y. , Shimizu, K. , Kasai, H. , Kitazato, H. , Fujiwara, Y. , Fujikura, K. , & Takishita, K. (2017). Comparison of morphological and DNA‐based techniques for stomach content analyses in juvenile chum salmon *Oncorhynchus keta*: A case study on diet richness of juvenile fishes. Fisheries Science, 83, 47–56.

[jfb70415-bib-0057] Scharf, F. S. , Buckel, J. A. , Juanes, F. , & Conover, D. O. (1997). Estimating piscine prey size from partial remains: Testing for shifts in foraging mode by juvenile bluefish. Environmental Biology of Fishes, 49, 377–388.

[jfb70415-bib-0058] Schooley, J. D. , Karam, A. P. , Kesner, B. R. , Marsh, P. C. , Pacey, C. A. , & Thornbrugh, D. J. (2008). Detection of larval remains after consumption by fishes. Transactions of the American Fisheries Society, 137, 1044–1049.

[jfb70415-bib-0059] Smith, B. E. , & Link, J. S. (2010). The trophic dynamics of 50 finfish and two squid species on the Northeast US Continental Shelf. NOAA Technical Memorandum NMFS‐NE‐21.

[jfb70415-bib-0060] Smith, K. M. , Byron, C. J. , & Sulikowski, J. A. (2016). Modeling predator–prey linkages of diadromous fishes in an estuarine food web. Marine and Coastal Fisheries, 8, 476–491.

[jfb70415-bib-0061] Stevens, J. R. , Jech, J. M. , Zydlewski, G. B. , & Brady, D. C. (2024). Response of estuarine fish biomass to restoration in the Penobscot River, Maine. Estuaries and Coasts, 47, 535–550.

[jfb70415-bib-0062] Stevens, J. R. , Saunders, R. , & Duffy, W. (2021). Evidence of life cycle diversity of river herring in the Penobscot River estuary, Maine. Marine and Coastal Fisheries, 13, 292–305.

[jfb70415-bib-0063] Thalinger, B. , Oehm, J. , Mayr, H. , Obwexer, A. , Zeisler, C. , & Traugott, M. (2016). Molecular prey identification in Central European piscivores. Molecular Ecology Resources, 16, 123–137. 26053612 10.1111/1755-0998.12436 PMC4744964

[jfb70415-bib-0064] Tollit, D. J. , Schulze, A. D. , Trites, A. W. , Olesiuk, P. F. , Crockford, S. J. , Gelatt, T. S. , Ream, R. R. , & Miller, K. M. (2009). Development and application of DNA techniques for validating and improving pinniped diet estimates. Ecological Applications, 19, 889–905. 19544732 10.1890/07-1701.1

[jfb70415-bib-0065] Toth, J. , Evert, S. , Zimmermann, E. , Sullivan, M. , Dotts, L. , Able, K. W. , Hagan, R. , & Slocum, C. (2018). Annual residency patterns and diet of *Phoca vitulina concolor* (Western Atlantic harbor seal) in a southern New Jersey estuary. Northeastern Naturalist, 25, 611–626.

[jfb70415-bib-0066] Traugott, M. , Thalinger, B. , Wallinger, C. , & Sint, D. (2021). Fish as predators and prey: DNA‐based assessment of their role in food webs. Journal of Fish Biology, 98, 367–382. 32441321 10.1111/jfb.14400 PMC7891366

[jfb70415-bib-0067] Valdez‐Moreno, M. , Quintal‐Lizama, C. , Gómez‐Lozano, R. , & García‐Rivas, M. d. C. (2012). Monitoring an alien invasion: DNA barcoding and the identification of lionfish and their prey on coral reefs of the Mexican Caribbean. PLoS One, 7, e36636. 22675470 10.1371/journal.pone.0036636 PMC3365883

[jfb70415-bib-0068] Ward, R. D. , Hanner, R. , & Hebert, P. D. N. (2009). The campaign to DNA barcode all fishes, FISH‐BOL. Journal of Fish Biology, 74, 329–356. 20735564 10.1111/j.1095-8649.2008.02080.x

[jfb70415-bib-0069] Waugh, J. (2007). DNA barcoding in animal species: Progress, potential and pitfalls. BioEssays, 29, 188–197. 17226815 10.1002/bies.20529

[jfb70415-bib-0070] Weber, D. C. , & Lundgren, J. G. (2009). Detection of predation using qPCR: Effect of prey quantity, elapsed time, chaser diet, and sample preservation on detectable quantity of prey DNA. Journal of Insect Science, 9, 41. 19619033 10.1673/031.009.4101 PMC3011838

[jfb70415-bib-0071] Weigt, L. A. , Driskell, A. C. , Baldwin, C. C. , & Ormos, A. (2012). DNA barcoding fishes. In W. J. Kress & D. L. Erickson (Eds.), DNA barcodes: Methods and protocols (pp. 109–126). Humana Press. 10.1007/978-1-61779-591-6_6 22684954

[jfb70415-bib-0072] Willis, T. V. , Wilson, K. A. , & Johnson, B. J. (2017). Diets and stable isotope derived food web structure of fishes from the inshore gulf of Maine. Estuaries and Coasts, 40, 889–904.

