# Supplementary material for: DNA‐based identification of anadromous fishes (Alosa spp., Family Clupeidae) in stomach contents of marine groundfish
Source: J Fish Biol. 2026 Mar 31;108(5):1614–26. doi: 10.1111/jfb.70415 (PMC13273095; doi:10.1111/jfb.70415)
Supplement: Supplementary file 1 — FIGURE S1. Pictures of Alosa pseudoharengus, Alosa aestivalis and Clupea harengus specimens representing the three digestion states (fresh, partial and well). Except for the fresh C. harengus, which is wild‐caught, all specimens were sampled from groundfish stomachs in the nearshore Gulf of Maine and positively identified to species using DNA barcoding in the present study. TABLE S1. Sequences of clupeid species sampled in the nearshore Gulf of Maine and their matching sequence record in the Barcode of Life Data System (BOLD). [file JFB-108-1614-s001.docx]

Supplemental Materials

**DNA-based identification of anadromous fishes (Alosa spp., Family Clupeidae) in stomach contents of marine groundfish**

Landon P. Falke, Stacy Rowe, Yuan Liu, Timothy F. Sheehan


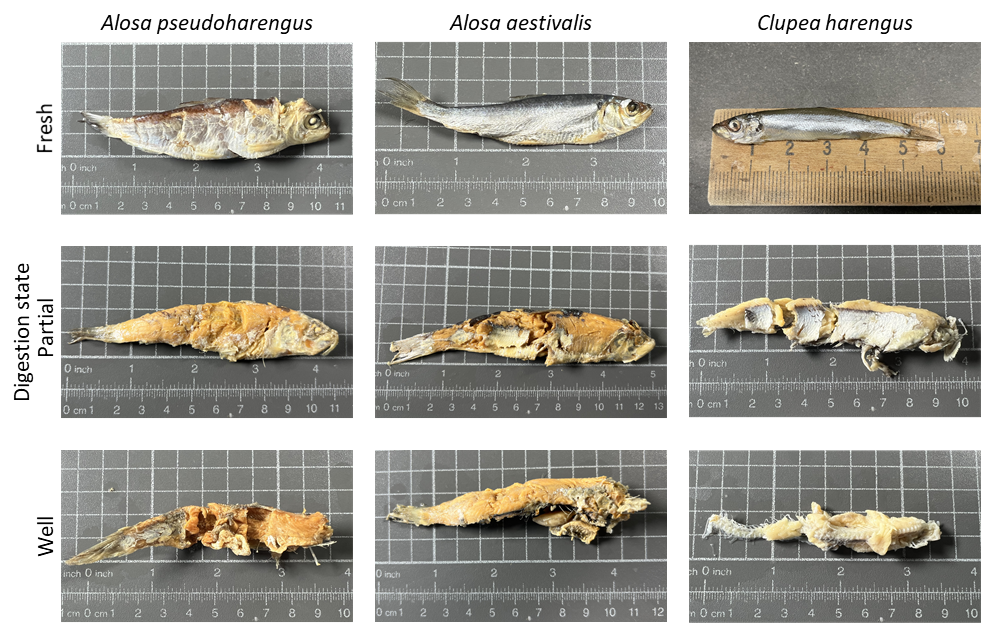


Figure S1. Pictures of *Alosa pseudoharengus*, *Alosa aestivalis*, and *Clupea harengus* specimens representing the three digestion states (fresh, partial, and well). Except for the fresh *C. harengus*, which is wild-caught, all specimens were sampled from groundfish stomachs in the nearshore Gulf of Maine and positively identified to species using DNA barcoding in the present study.

Table S1. Sequences of clupeid species sampled in the nearshore Gulf of Maine and their matching sequence record in the Barcode of Life Data System (BOLD).

| Scientific name | Common name | Percent identity to reference sequence | | BOLD match sequence record # | Amplicon sequence |
| --- | --- | --- | --- | --- | --- |
| *Alosa aestivalis* | Blueback Herring | 100 | SCFAC616-06 | | CAACYAATCAYAAAGATATYGGCACCCTTTACCTAGTATTTGGTGCCTGAGCAGGGATAGTAGGCACTGCCTTAAGTCTCTTAATCCGAGCAGAACTGAGCCAACCCGGGGCACTTCTCGGAGACGATCAGATCTATAACGTCATCGTTACGGCGCACGCCTTCGTAATAATCTTCTTCATAGTAATGCCAATTCTAATTGGTGGCTTTGGGAACTGACTAGTCCCCCTTATGATCGGGGCACCAGACATGGCATTCCCGCGAATGAACAACATGAGCTTCTGACTACTTCCGCCCTCATTCCTCCTTCTCCTTGCCTCATCCGGGGTTGAGGCCGGGGCAGGAACCGGATGAACAGTCTACCCGCCCTTGGCAGGCAATCTTGCCCACGCCGGAGCGTCCGTCGATCTAACTATCTTCTCTCTCCATCTAGCAGGTATCTCATCAATTCTTGGGGCCATTAATTTTATTACCACAATTATTAATATGAAACCCCCTGCAATCTCACAATATCAAACACCCCTATTCGTGTGATCCGTGCTCGTAACGGCCGTTCTCCTTCTTCTCTCACTCCCTGTGTTAGCTGCTGGGATTACAATGCTCCTAACAGACCGAAATCTAAATACGACCTTCTTTGACCCGGCAGGGGGAGGGGACCCAATTCTATATCAACACCTATTCTGATT |
| *Alosa pseudoharengus* | Alewife | 100 | BCF572-07 | | TCAYAAAGATATYGGCACCCTTTACCTAGTATTTGGTGCCTGAGCAGGGATAGTAGGCACTGCCTTAAGTCTCTTAATCCGAGCAGAACTGAGCCAACCCGGGGCACTTCTCGGAGACGATCAGATCTATAACGTCATCGTTACGGCGCACGCCTTCGTAATAATCTTCTTCATAGTAATGCCAATTCTAATTGGTGGCTTTGGGAACTGACTAGTCCCCCTTATGATCGGGGCACCAGACATGGCATTCCCACGAATGAACAACATGAGCTTCTGACTACTTCCGCCCTCATTCCTCCTCCTTCTTGCCTCATCCGGGGTTGAGGCCGGGGCAGGAACCGGATGAACAGTCTACCCGCCCTTGGCAGGTAATCTTGCCCACGCCGGAGCGTCCGTCGATCTAACTATCTTCTCTCTTCATCTAGCAGGTATCTCATCAATTCTTGGGGCCATTAATTTTATTACCACAATTATTAATATGAAACCCCCTGCAATCTCACAATATCAAACACCCCTATTTGTGTGATCCGTGCTTGTAACGGCCGTTCTCCTTCTTCTCTCACTCCCTGTGTTAGCTGCTGGGATTACAATGCTCCTAACAGACCGAAATCTAAATACGACCTTCTTTGACCCGGCAGGGGGAGGGGACCCAATTCTATATCAACACCTATTCTGATT |

Table S1 continued

| Scientific name | Common name | Percent identity to reference sequence | | BOLD match sequence record # | Amplicon sequence |
| --- | --- | --- | --- | --- | --- |
| *Alosa sapidissima* | American Shad | 99.3 | ANGBF34595-19 | | CAGGTCAACYAATCAYAAAGATATCGGCACCCTTTACCTAGTATTTGGTGCCTGAGCAGGGATAGTAGGCACTGCCTTAAGTCTCTTAATCCGAGCCGAACTGAGCCAACCCGGGGCGCTTCTCGGAGATGATCAGATCTATAACGTCATCGTTACGGCGCACGCCTTCGTAATAATCTTCTTCATAGTAATGCCAATTCTAATTGGCGGCTTTGGGAATTGACTGGTCCCCCTTATGATCGGGGCACCAGACATGGCATTCCCACGAATGAACAACATGAGCTTCTGACTACTTCCACCCTCATTCCTCCTCCTCCTTGCCTCCTCCGGAGTTGAGGCCGGGGCAGGAACCGGGTGAACAGTCTACCCACCTTTGGCAGGCAATCTTGCCCACGCCGGAGCATCCGTCGACCTAACTATCTTCTCTCTTCATCTAGCAGGTATTTCATCAATTCTTGGGGCCATTAATTTTATTACCACAATCATTAATATGAAACCCCCTGCAATTTCACAATATCAAACACCCCTATTTGTGTGATCCGTGCTTGTAACGGCCGTTCTCCTTCTTCTCTCACTCCCTGTGCTAGCTGCTGGGATTACAATGCTCCTAACAGACCGAAATCTAAATACAACCTTCTTTGACCCGGCAGGGGGAGGGGACCCAATTTTATATCAACACCTATTCTGATT |
| *Brevoortia tyrannus* | Atlantic Menhaden | 100 | GBGCA10270-15 | | GCGGATAACAATTTCACACAGGTCAACYAATCAYAAAGATATYGGCACCCTCTACCTGGTATTTGGTGCCTGAGCAGGGATGGTAGGCACTGCCTTAAGTCTCTTAATCCGAGCAGAACTGAGCCAACCCGGGGCACTTCTCGGAGACGATCAGATCTATAATGTTATCGTTACGGCGCACGCCTTCGTAATAATCTTCTTCATAGTAATGCCAATTCTAATTGGCGGCTTTGGGAACTGACTAGTCCCCCTTATGATCGGGGCACCAGACATGGCATTCCCGCGAATGAACAACATGAGCTTCTGACTCCTTCCCCCCTCATTTCTCCTCCTCCTTGCCTCATCCGGAGTTGAAGCGGGAGCAGGGACCGGATGAACAGTCTACCCACCTTTGGCAGGCAATCTAGCTCACGCCGGAGCATCAGTTGATCTAACTATTTTCTCCCTCCACCTAGCAGGTATTTCATCTATTCTTGGAGCCATTAATTTCATTACTACAATTATTAACATGAAACCCCCTGCAATTTCACAATACCAAACGCCGTTATTTGTATGATCCGTGCTTGTAACAGCTGTTCTTCTTCTCCTTTCACTCCCTGTCTTAGCTGCTGGAATTACAATACTGCTGACAGACCGAAATCTTAATACGACCTTCTTCGACCCGGCAGGAGGAGGAGATCCAATTTTATATCAACACCTGTTCTGATTYTTYGGYCACCCRGAAGTACTGGCCGTCGTTTTA |

Table S1 continued

| Scientific name | Common name | Percent identity to reference sequence | | BOLD match sequence record # | Amplicon sequence |
| --- | --- | --- | --- | --- | --- |
| *Clupea harengus* | Atlantic Herring | 100 | FOAD058-05 | | GCGGATAACAATTTCACACAGGTCAACYAATCAYAAAGATATYGGCACCCTTTACCTAGTATTTGGTGCCTGAGCAGGAATGGTGGGCACAGCCCTAAGTCTCCTAATCCGTGCAGAACTTAGCCAACCTGGGGCTCTCCTTGGAGACGACCAGATCTATAATGTTATTGTTACTGCACATGCCTTCGTAATAATTTTCTTTATAGTAATGCCGATTCTAATTGGAGGGTTTGGAAACTGACTAATTCCTCTTATGATCGGAGCGCCAGATATGGCATTCCCTCGAATAAACAATATGAGCTTCTGACTACTTCCCCCCTCATTCCTCCTACTACTAGCCTCCTCCGGAGTTGAAGCCGGGGCGGGGACCGGGTGAACGGTATATCCTCCTCTGTCAGGAAATCTGGCCCATGCAGGAGCATCAGTTGACCTAACCATTTTTTCACTTCATCTAGCAGGTATTTCCTCTATTCTAGGGGCCATTAATTTCATTACCACAATTATTAATATGAAACCACCCGCAATCTCACAATACCAAACGCCTCTGTTTGTCTGATCCGTTCTTGTTACAGCTGTTCTACTTCTTCTATCGCTGCCTGTGCTAGCTGCCGGAATTACAATGCTTCTTACAGATCGAAA |
